# Identification of cancer sex-disparity in the functional integrity of p53 and its X chromosome network

**DOI:** 10.1038/s41467-019-13266-3

**Published:** 2019-11-26

**Authors:** Sue Haupt, Franco Caramia, Alan Herschtal, Thierry Soussi, Guillermina Lozano, Hu Chen, Han Liang, Terence P. Speed, Ygal Haupt

**Affiliations:** 1 Tumor Suppression Laboratory, Peter MacCallum Cancer Centre, 305 Grattan St, Melbourne, Victoria 3000 Australia; 2 Sir Peter MacCallum Department of Oncology, The University of Melbourne, Parkville, Victoria 3010 Australia; 3 Department of Biometrics Novotech, Carlton, Victoria 3053 Australia; 4 Department of Oncology-Pathology, Karolinska Institute, Cancer Center Karolinska, Solna, Sweden; 5 INSERM, U1138, Centre de Recherche des Cordeliers, Paris, France; 6 The University of Texas, MD Anderson Cancer Center, Houston, TX 77030 USA; 7 Graduate Program in Quantitative and Computational Biosciences, Baylor College of Medicine, Houston, TX 77030 USA; 8 Department of Bioinformatics and Computational Biology, The University of Texas MD Anderson Cancer Center, Houston, TX 77030 USA; 9 Department of Systems Biology, The University of Texas MD Anderson Cancer Center, Houston, TX 77030 USA; 10 Bioinformatics Division, The Walter and Eliza Hall Institute of Medical Research, Parkville, Victoria 3052 Australia; 11 Department of Mathematics and Statistics, University of Melbourne, Parkville, Victoria 3010 Australia; 12 Department of Clinical Pathology, University of Melbourne, Parkville, Victoria 3010 Australia; 13 Department of Biochemistry and Molecular Biology, Monash University, Melbourne, Victoria Australia

**Keywords:** Cancer genetics, Tumour-suppressor proteins, Computational biology and bioinformatics

## Abstract

The disproportionately high prevalence of male cancer is poorly understood. We tested for sex-disparity in the functional integrity of the major tumor suppressor p53 in sporadic cancers. Our bioinformatics analyses expose three novel levels of p53 impact on sex-disparity in 12 non-reproductive cancer types. First, *TP53* mutation is more frequent in these cancers among US males than females, with poorest survival correlating with its mutation. Second, numerous X-linked genes are associated with p53, including vital genomic regulators. Males are at unique risk from alterations of their single copies of these genes. High expression of X-linked negative regulators of p53 in wild-type *TP53* cancers corresponds with reduced survival. Third, females exhibit an exceptional incidence of non-expressed mutations among p53-associated X-linked genes. Our data indicate that poor survival in males is contributed by high frequencies of *TP53* mutations and an inability to shield against deregulated X-linked genes that engage in p53 networks.

## Abstract (web summary)

There is disproportionally high cancer prevalence in males. Here, the authors analyse the tumour suppressor p53 in sporadic cancers, highlighting a higher incidence of its mutation in males. Males are further disadvantaged by a failure to shield against the expression of damaged X-linked genes in p53-networks. These factors likely contribute to sex-disparity.

## Introduction

Cancer incidence and death rates are higher in males than females^1^, and despite extensive genome-wide analyses (e.g., refs. ^2,3^), the explanation for this inequity is incomplete. Cancer sex disparity is evident across multiple non-reproductive, sporadic cancers^1^. Male lifestyle has been blamed for greater exposure to carcinogens^4^, however, it is likely that both environmental and genetic factors influence cancer incidence. Emerging molecular and physiological peculiarities that appear to affect disparity in animal studies, prompted NIH to demand preclinical evaluation in both males and females^5^. Intriguingly, not all animals develop cancer, and in elephants, protection of both sexes is attributed to multiple copies of the *TP53* gene^6^. The lack of cancer in elephants also argues that p53 can override an impact of hormones on cancer sex disparity. Critically, the single *TP53* gene copy is outstanding as the most commonly altered gene in human cancers^7^. Further, in a compound mutant p53 mouse model that we generated, males developed more aggressive cancers and reduced lifespan than females^8^. This triggered us to investigate the connection between p53 and cancer sex disparity in non-reproductive cancers.

Tumour suppressor p53 is a transcription factor that crucially integrates stress signals into protective cellular responses. Stresses such as DNA damage, trigger activating-modifications in p53^9^, which promote temporary arrest to facilitate repair, or permanent growth inhibition, including senescence, apoptosis^10^ and ferroptosis^11^. P53 also targets core-regulatory molecules in metabolic pathways (glycolytic, lipid and nucleic acid^10,12^), immune responses^13,14^ and aneuploidy^15^. Remarkably, the activities attributed to p53, include the four key processes recently identified through gene expression analyses to link to cancer sex disparity: (1) immune response, (2) apoptosis and cell cycle, (3) metabolism-related and (4) DNA-repair and p53-pathways (using standard autosome pathways of the gene set enrichment analysis (GSEA)^2^. In healthy individuals, inherited genetic variants in the standard p53-pathways were more frequent than for all other pathways and linked to cancer exclusively, and not other diseases^16^. These findings highlight the importance of p53 and its autosome partner proteins as the greatest natural deterrent against cancer. Whether these p53 functions are distinct between cancers in males and females remain unanswered.

P53 activities and expression are normally tightly regulated, and a universal signature of human cancers is the loss of effective p53 tumour-suppressor activity^17^. Breakdown in p53 function is attributed almost equally to *TP53* gene mutation^7^, or excessive levels of its key negative regulators: MDM2 and MDM4^18^. *TP53* mutation not only strips away its tumour-suppressive capacities^19^ but also confers neomorphic, cancer-promoting properties, referred to as gain-of-function (GOF; reviewed^20^). Discrepant rates of cancer and related mortality between males and females provoked us to investigate whether the tumour-suppressive ability of p53 is equally competent between the sexes. In this study of non-reproductive cancers, we identified three novel layers of risk for cancer sex disparity that are critically affected by p53 status and subject to the function and expression of its X-chromosome interactors.

## Results

### Sex disparity in *Tp53* mutation frequency in US population

The most common somatic cancers are more frequent in males^21^. To explore this disparity, we examined whether mutation of the tumour-suppressor gene *TP53* differs in frequency in the human population, between male and female somatic cancers. This human sex-disparity study was prompted by our discovery of a link between p53-functionality and cancer aggression in male mice^8^.

Directly measuring the relative general population cancer risk for *TP53* mutation between males and females is currently not feasible as: (1) it is an age-dependent parameter, which requires sampling across multiple age-brackets; (2) sampling every organ is impractical and (3) even with the advances in detecting circulating tumour DNA, the current technology lacks sufficient sensitivity.

**Fig. 1:**
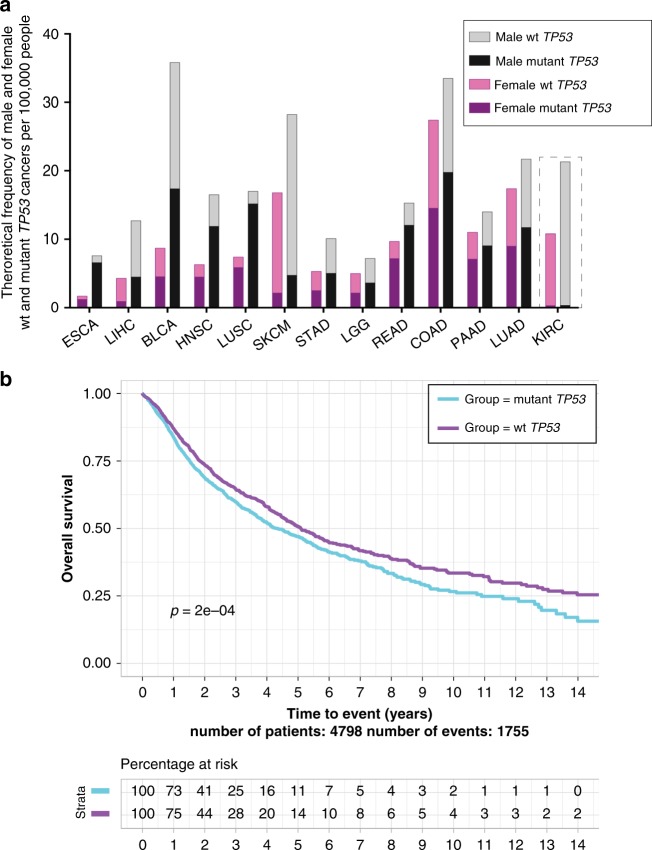
The frequency of cancer with *TP53* mutation is greater for males than females in multiple non-reproductive cancer types with *TP53* mutation corresponding to poorest survival. **a** Predicted *TP53* mutation incidence in male and female cancers in the US population as calculated from combined SEER and TCGA data for the13 most abundant non-reproductive cancers: oesophageal carcinoma (ESCA), liver hepatocellular carcinoma (LIHC), bladder urothelial carcinoma (BLCA), head and neck squamous cell carcinoma (HNSC), lung squamous cell carcinoma (LUSC), skin cutaneous melanoma (SKCM), lower grade glioma (LGG), rectum adenocarcinoma (READ), stomach adenocarcinoma (STAD), colon adenocarcinoma (COAD), pancreatic adenocarcinoma (PAAD) and lung adenocarcinoma (LUAD and the exception (demarcated by a dashed box), kidney renal clear cell carcinoma (KIRC). **b** Overall survival (OS) across 14 years, for patients from the 12 disparity cancers in TCGA is stratified for cancers with wt *TP53* (aquamarine) and mutant *TP53* (turquoise).

Our alternative approach was to amalgamate two complementary data sets to compare *TP53* mutation incidence between male and female cancers. First, the National Cancer Institute’s Surveillance, Epidemiology, and End Results program (SEER data^21^) provides the comprehensive cancer incidence for the overall US population. This cannot be obtained from the second data set, The Cancer Genome Atlas (TCGA^22^). Rather, TCGA offers the rates of cancer-associated *TP53* mutations (or pathogenic mutations) from random cancer sampling across the sexes, but does not claim to be representative of population incidence. The pathogenic definition is built on the observation that most cancer-associated *TP53* mutations occur in its DNA-binding domain and cause interruption of p53 function^10^ (as defined in detail in the Methods section). From TCGA, we considered all *TP53* exome mutations with functional impact to be pathogenic. Specifically, we examined our hypothesis that *TP53* mutation frequency is distinct between the sexes by applying a probability calculation for 13 non-reproductive cancers. Explicitly, this represents all the cancers with at least five TCGA cases in all four groups: female mutant *TP53*, female wild-type (wt) *TP53*, male mutant *TP53* and male wt *TP53*. Our calculations show for the first time that males have a higher probability of developing cancers with *TP53* mutations than females, for the majority of non-reproductive cancers: subsequently referred to as the 12 disparity cancers (Fig. 1a; Table 1; Supplementary Table 1). This finding forms the focus of this study. Kidney (KIRC), which has a very low *TP53* mutation incidence and originates from distinct developmental tissue^23^, indicates that our finding cannot simply be explained as an obvious direct correlation between cancer prevalence and *TP53* mutation.

**Table 1:** Frequencies of cancers and *TP53* mutation by sex.

Cancer type	Sex	Total patients by disease (TCGA)^a^	Patients with *TP53* mutation (TCGA)	Frequency of *TP53* mutation per cancer (TCGA)	Frequency of cancer per 100,000 (SEER)^b^	Frequency of *TP53* mutation per 100,000 (SEER & TCGA)	Ratio: M/F frequency of cancer with *TP53* mutation in the US population
ESCA	F	27	19	0.70	1.7	1.2	5.5
	M	158	137	0.87	7.6	6.6	
LIHC	F	121	26	0.21	4.3	0.9	4.9
	M	252	89	0.35	12.7	4.5	
BLCA	F	108	56	0.52	8.7	4.5	3.9
	M	303	147	0.49	35.8	17.4	
HNSC^c^	F	138	98	0.71	6.3	4.5	2.7
	M	371	267	0.72	16.5	11.9	
LUSC	F	129	102	0.79	7.4	5.9	2.6
	M	362	323	0.89	17	15.2	
SKCM	F	179	23	0.13	16.8	2.2	2.2
	M	286	48	0.17	28.2	4.7	
STAD	F	157	74	0.47	5.3	2.5	2.0
	M	280	139	0.50	10.1	5.0	
LGG	F	227	98	0.43	5	2.2	1.7
	M	281	141	0.50	7.2	3.6	
READ	F	77	57	0.74	9.7	7.2	1.7
	M	89	70	0.79	15.3	12.0	
COAD	F	215	114	0.53	27.4	14.5	1.4
	M	234	138	0.59	33.5	19.8	
PAAD	F	79	51	0.65	11	7.1	1.3
	M	102	66	0.65	14	9.1	
LUAD	F	277	143	0.52	17.4	9.0	1.3
	M	239	129	0.54	21.7	11.7	
KIRC	F	179	5	0.03	10.8	0.3	1.1
	M	314	5	0.02	21.3	0.3	

*M* male, *F* female

^a^TCGA data^22^

^b^SEER data^21^

^c^SEER data for oral cavity and pharynx

To examine the functional impact of *TP53* mutations in the TCGA cohort, we analysed TCGA for overall cancer patient survival, for the 12 disparity cancers across 14 years. Together, patients with mutated *TP53* from these 12 disparity cancers had significantly poorer survival outcomes than their wt *TP53* counterparts (Log-rank test; *p*-value = 0.0002, Fig. 1b). It is relevant to add that this finding was validated recently in a study of individual cancers, where nine of the cancers in this study showed poorer survival for patients with *TP53* mutation, while individual data analyses for the other three cancers were not included^24^. Given our finding that *TP53* mutations occur at higher frequency in males than in females in the 12 disparity cancers, we expect *TP53* mutational status to contribute significantly to the higher incidence of cancer deaths in males in the general population, over females.

This defines our first cornerstone that: *TP53* mutation frequency is higher among US males than females for the majority of non-reproductive cancers, corresponding with poorest survival outcomes for these cancers.

### X chromosome encodes many proteins that interact with p53

The normal strict control of the tumour suppressor p53 and its multiple downstream effector pathways (reviewed in ref. ^25^) are disrupted in cancer, either by *TP53* mutation or by malfunction in this p53 network. The possibility that the p53 network is not equally functional between males and females is unexplored. We chose to search for connections between p53 and the X chromosome because: (1) numerous tumour-suppressor genes are encoded on the X chromosome^26^, but are under-studied for connection to p53 and sex disparity; (2) X-chromosome genes are not yet incorporated into established p53 networks (eg. KEGG^16^); (3) X-chromosome analysis is frequently avoided in sporadic diseases because X-inactivation (Xi) silencing in females complicates data interpretation (reviewed in refs. ^27,28^) and (4) males are at greater disease risk than females from X-chromosome mutations (reviewed in ref. ^29^).

To identify coding genes that interact with p53 on the X chromosome^28,30^, we adopted the unbiased Search Tool for the Retrieval of Interacting Genes/Proteins (STRING) database tool^31^. A reproducible p53-STRING set of 90 genes was defined (with confidence limits ≥ 0.3) from among the ~800 coding genes on the X chromosome (Supplementary Data 1, TAB1; Supplementary Fig. 1a). We note that due to the rapid pace of new discoveries, some partnerships remain to be captured. Among the STRING set are key p53 modifiers (kinases, E3-ligases and a de-ubiquitinases). Gene Ontology (GO) analysis shows that the p53-STRING set is rich in fundamental p53 regulatory processes, including regulation of apoptosis, cell cycle, DNA structural integrity and hypoxic response. Additional notable processes included are regulation of protein acetylation and histone modifications, among others (Supplementary Fig. 1b; with analyses shown for both the inclusion, or not, of *TP53*, respectively, Supplementary Data 1, TAB2, 3). The specific example of the p53-negative regulator HUWE1 supports this link, where reduction of its levels corresponds with diminished cell growth only in cells with wt *TP53*, but not mutant (among 135 lines of the Achilles project, Supplementary Fig. 1c); consistent with the relief of wt p53 from constraints resulting in growth inhibition.

### X-chromosome exome mutations are abundant in male cancers

**Fig. 2:**
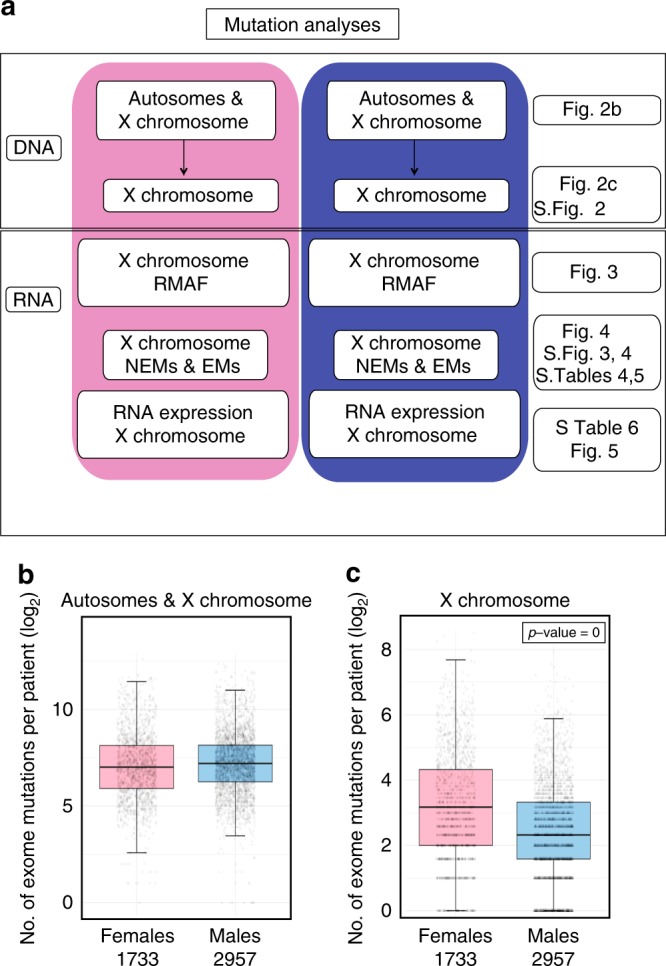
Frequency of X-chromosome mutation was greater in females than males for the 12 disparity cancers. **a** The scheme shows our strategy for bioinformatic data analyses of exome mutations, their expression and overall mRNA content analyses in TCGA data sets; with corresponding figure identity listed on the right. On a per patient basis, (**b**) the number of exome mutations in the autosomes and X chromosomes and the (**c**) number of exome mutations for the X chromosome alone are presented (and importantly this collective data aligns with the individual cancers as evident in Supplementary Fig. 2a, b). Females are indicated in pink and males in blue. These are standard boxplots with the median as the centre line across the boxed-in upper and lower quartiles, with maximum and minimum values demarcated by whiskers.

To search for evidence of sex disparity in the p53-STRING set, we analysed TCGA data for somatic exome mutations (DNA) and their expression (mRNA); as outlined in Fig. 2a (with details included in the Methods section). To adjust for potential confounding factors in our statistical tests, we applied propensity scores to the analyses as described by Yuan et al.^2^. Patient data were controlled for the key parameters of age, race, smoking history and tumour stage (as detailed in the Methods). At the whole-exome level, only minor differences in the total number of gene mutations per patient (determined for 1733 females and 2757 males; using the Poisson log linear model, 95% CI [1.01, 1.02], *p*-value ≈0) were evident between the sexes in the disparity cancers, excluding the Y chromosome (Fig. 2b). In contrast, separate analysis of the shared allosome, the X chromosome, revealed a higher incidence (≈ twofold) of exome mutations in cancers of females than males (95% CI [1.86, 1,92], *p*-value ≈ 0). This can be seen for all cancers together in Fig. 2c and for each cancer individually in Supplementary Fig. 2a. This was also evident for the p53-STRING set for the disparity cancers together (95% CI [1.55, 1.72], *p*-value ≈0; Supplementary Data 2, TAB1–7; and shown also for each cancer individually in Supplementary Fig. 2b). This is in accord with there being two X chromosomes in females, and one in males per chromosome set. Xi during development of a single female X chromosome ensures single-allele expression of most X-linked genes in females. This effectively evens X-chromosome gene expression between the sexes. Exceptions to this in females are the limited ~15% of escape-genes^32^, which are largely pseudoautosomal (reviewed in ref. ^33^) with a few exceptions^3^. The identity of the X-chromosome copy that is silenced by Xi cannot be deduced from TCGA exome data. Equally, the X-chromosome gene mutations that are expressed cannot be identified from exome sequencing. To identify which X-chromosome exome mutations were expressed in male and female cancers, it was necessary to search for the corresponding mutations in their mRNA.

### Elevated X-chromosome RNA-mutated allele frequency in males

Variant allele frequency (VAF) is a commonly used metric employed to identify  somatic variants at the DNA level. To date, a measure for RNA expression of DNA mutations has not been formally assigned. To fill this gap, we created a novel concept: RNA-mutated allele frequency (RMAF), which we define as the mRNA equivalent of VAF. Explicitly, RMAF is a measure of the relative frequency with which a mutation in DNA is expressed in mRNA, relative to the total expressed mRNA (refer to Methods).

We predicted that for the X chromosomes, the RMAF of females would be lower than for males. This was founded on the expectation that for males, all mutations from their single X chromosome can be expressed. For healthy females, expectation of lower RMAF was founded on the prediction that mutations are evenly spread across the two individual X chromosomes, and that X inactivation is completely random. However, in contrast to healthy females, in cancers we could not presume that this would be the case. This led us to formerly test RMAF across the 12 disparity cancers for females and males.

**Fig. 3:**
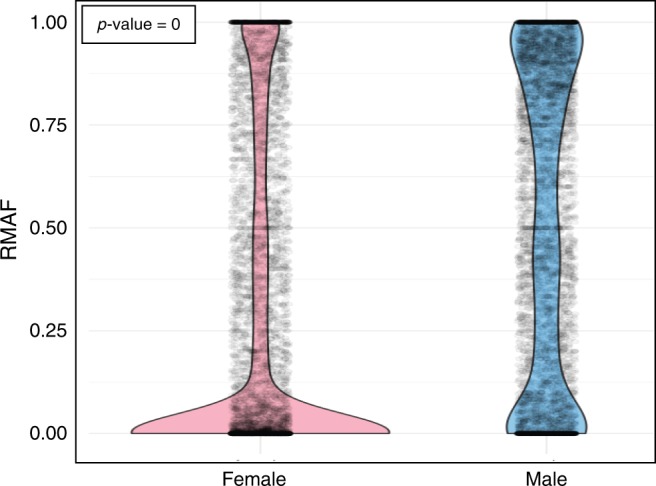
RNA-mutated allele frequency (RMAF) for X-chromosome genes was particularly high in male cancers, while in female cancers most mutations were not expressed. RMAF was calculated for the 12 disparity cancers, for females and males independently (and pertinently this combined data reflects the individual cancers as evident in Supplementary Fig. 2c). RMAF was quantified using a scale between 0 and 1. For a specific exome nucleotide mutation, 0 indicates the complete absence of mRNA for this particular mutation, where the gene is expressed at the mRNA level; 1 defines mutation in this nucleotide in the corresponding mRNA in 100% of cases. Females are indicated in pink and males in blue.

We found that in female cancers, more than half of their X-chromosome exome mutations were not transcribed into their corresponding mRNA (4002/7416; 54% RMAF = 0, median 0). This is displayed for the 12 disparity cancers altogether in Fig. 3, and for each cancer individually in Supplementary Fig. 2c. In contrast, as we predicted, the majority of exome mutations were transcribed in the mRNA of male cancers (4304/5335; 82% RMAF > 0, median 0.58). Comparing male and female RMAF at the individual mutation level, adjusted for cancer type, on the logit scale, we found female RMAF was indeed significantly smaller than male RMAF (*p*-value ≈ 0). To tease this further apart, we interrogated which specific gene mutations were expressed or not, in male and female cancers.

These discoveries define our second cornerstone that: the X chromosome encodes vital protein regulators of genomic fidelity that are linked to p53 function; and males are at higher risk of expressing mutations of these genes due to their obligatory mono-allelic expression, in contrast to the chromosomal choice in females.

### Restricted expression of X-linked gene mutations in females

Given the lower incidence of non-reproductive cancers in females, we hypothesised that they are preferentially protected from the expression of cancer-risk gene mutations, compared with males. As we were unable to test this in healthy people, we examined data from cancer biopsies, aware that we were searching for remnants of a broken protective system. We compared the expression of X-chromosome mutations between cancers of females and males, where X-chromosome exome mutations that were not detected at the mRNA level (RMAF ≤ 0.20; Fig. 3) are referred to as non-expressed mutations (NEMs, see the Methods section).

**Fig. 4:**
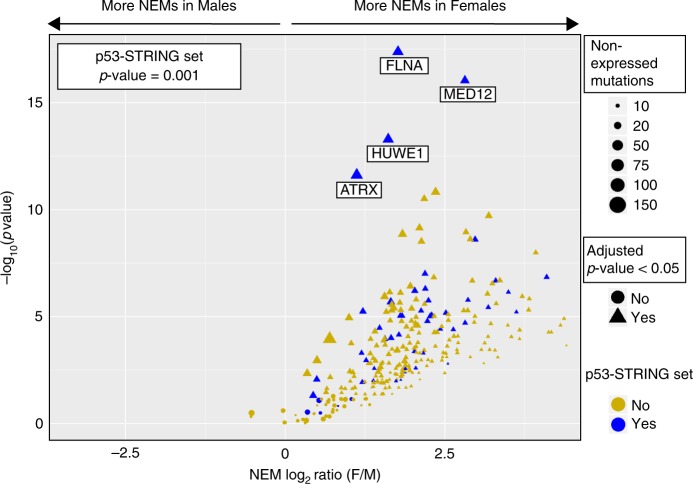
Non-expressed mutation (NEM) frequencies were significant only in females, and the p53-STRING set genes were more prominent than the other X-chromosome genes. The relative incidence (*x*-axis: log_2_ ratio [F/M]) and significance [*y*-axis; - log_10_(p-value)] of NEMs was compared for all X-linked genes between females and males of the 12 disparity cancers. P53-STRING set genes are blue, others are gold. NEMs showing a significant sex difference are indicated by a triangle, and those lacking significance by a circle. The number of NEMs for each gene are indicated by a correlative, sliding scale of size.

The incidence of NEMs reached significance only in females (Fig. 4; Weighted chi-square test, Benjamini–Hochberg adjusted *p*-value ≤ 0.05) only in females.

In the disparity cancers, mutations in 268 genes (~1/3 of the X-linked coding genes) were significantly less expressed in cancers of females than males. GO analysis of these 268 genes identified pathways of protein acetylation (including histone acetylation); histone modification and negative regulation of both organelle organisation and microtubule depolymerisation (Supplementary Data 3, TAB1–3; Supplementary Fig. 3a). If the double load of X-chromosome exome mutations in females (Supplementary Data 2) were equally distributed across the active and inactive X-chromosome genes on a random basis, a greater number of NEMs would be expected in female cancers distributed as a function of gene size, simply due to Xi. Importantly, we did not find significant correlation between X-gene sizes and NEMs (Pearson coefficient: *r* = 0.3, data not shown).

Strikingly, our analysis revealed an unexpectedly high incidence of NEMs in the p53-STRING gene set (Wilcoxon rank test *p*-value = 0.001 Supplementary Data 3, TAB4, 5). The most significant NEMs in these cancers occurred in the p53-STRING genes *FLNA*, MED12, *HUWE1, ATRX* (Fig. 4). In total, over half the p53-STRING genes (50 out of 90) were identified with a significantly disproportionate NEM incidence  between males and females (adjusted *p*-value were ≤ 0.05). GO analysis of these 50 genes identified their involvement in fundamental pathways: histone modifications (ubiquitination, H3–H9 modification and trimethylation), protein localisation (to chromosome and telomeric region), regulation of telomere maintenance, and cell cycle DNA replication as key pathways (Supplementary Data 3, TAB6; Supplementary Fig. 3b). The functional association of these 50 genes with p53 was confirmed by extensive literature mining (Supplementary Data 3, TAB7). Of note, analysis of the KIRC data set did not identify significant NEMs for the X chromosome (Supplementary Data 3, TAB8). However, analyses of larger KIRC data sets will be required for validation. Overall, this exceptional incidence of NEMs among these p53-STRING genes supports our hypothesis that resistance to cancers of the disparity set is conferred in females through selective protection of the p53-X-chromosome network. While NEMs are relevant to cancer protection, it is the expressed mutations that pose cancer risk.

This defines our third cornerstone that compared with males, females express a significantly lower proportion  of their X-linked gene mutations, and this is outstanding for the p53-STRING set.

### Frequent expression of X-linked gene mutations in cancers

In male and female cancers, X-chromosome expressed mutations (EMs, RMAF ≥ 0.75, see Methods) were numerous (Supplementary Data 4, TAB1). Consistent with the relevance of the p53-STRING set in cancer, these genes were among those with the most frequent EMs. Specifically, more than half of the p53-STRING set genes (47) were among the 299 genes with EMs in female cancers (Supplementary Data 4, TAB2,3)﻿. Similarly, in male cancers, 53 genes of the p53-STRING set were among the 341 genes with EMs (Supplementary Data 4, TAB4,5). This abundance of p53-STRING set EMs in males and females supports the disruption of p53 networks in cancers of both sexes (Supplementary Data 4, TAB6)﻿. GO analysis of the EM genes in females (299) and males (341) identified distinct pathway vulnerabilities between the sexes (Supplementary Fig. 4a, b, respectively; Supplementary Data 4, TAB7). In females, EM genes were prevalent in processes associated with internal amino acid acetylation and covalent chromatin modification, while in males the processes of glycosyl compound metabolic process, histone modification and response to iron(III) ion were pronounced.

### X-linked inhibitors of wt p53 are highly expressed in cancer

To examine whether altered gene expression levels of the p53-STRING set are evident in the disparity cancers, we adopted the comparative expression analysis tool GSEA. This computational approach was applied to measure statistically significant differences in the expression of a designated list of genes, between two related populations. Enrichment in gene expression was quantified according to a normalised enrichment score (NES), and statistical significance was defined by an adjusted *p*-value of <0.25.

We chose to focus on the X-linked negative regulators of wt p53. We postulated that high expression of these negative regulators could reduce wt p53 activity, in an analogous manner to high levels of the MDM proteins, which have proven to be oncogenic (reviewed in ref. ^34^). Literature mining led us to identify 11 negative regulators of wt p53 among the p53-STRING set: UBE2A (RAD6^35^), MAGEA2^36^ and UTP14A^37^, CDK16^38^, PPEF1^39^, HUWE1^40^, CUL4B^41^, DDX53^42^, NOX1^43^, PMSD10^44^ and TAF1^45,46^. We tested the relative expression levels of these genes in tumours and their matched normals, for each cancer type separately, with additional stratification according to wt *TP53* status and patient sex. We undertook these studies on 11 of the 12 disparity cancers, but excluded LGG, due to a lack of matched normal tissue.

**Fig. 5:**
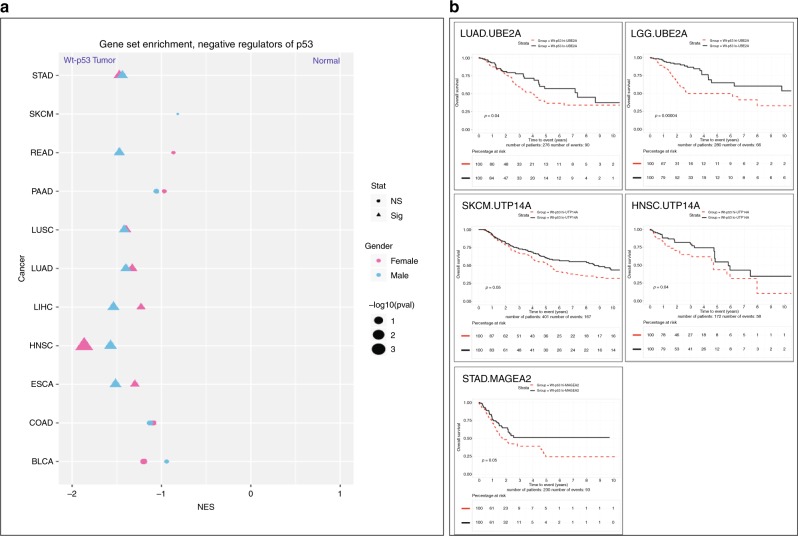
High expression levels of negative regulators of p53 among the p53-STRING set are more frequent in wt *TP53* cancers and associate with reduced survival. **a** Gene set enrichment analysis (GSEA) of the expression of the X-linked negative regulators of p53 compared between the disparity cancers with wt *Tp53* and their matched normals. **b** Kaplan–Meier survival curves were generated for cancer patients with wt *Tp53* tumours on the basis of the relative expression levels of the X-linked p53-negative regulators UBE2A, UPT14A and MAGE2A, respectively (top median: broken red line and bottom median: solid black line).

Expression of these genes was evidently higher in all the 11 cancers compared with their matched normals (Fig. 5a displays graphically the results of the analyses that are recorded in Supplementary Data 5, TAB1). Notably, this correlation was significant in males for seven of the cancers and for six in females. Even where significance fell below the designated threshold, there was a universal trend for enhanced expression in the tumours over the normals. It is appropriate to emphasise that this analysis ranks the expression of each gene individually for each cancer separately, relative to the expression of all other X-linked genes in that specific cancer type.

Adopting three negative regulators as specific examples, (as an extension of our rational candidate approach), we tested whether their expression levels could be linked to the measurable biological outcome of survival in cancers with wt *TP53*. We examined survival duration over 10 years, relative to the individual expression of *UBE2A* (which stimulates p53 degradation by MDM2^35^), *MAGEA2* (which inhibits p53 acetylation^47^; prevents senescence induction and promotes proliferation^36^) and *UTP14A* (which binds p53 and promotes its degradation^37^) for each cancer, in a wt *TP53* context. In this individual cancer approach, we did not further stratify for patient sex to avoid diminishing statistical power, which is a risk associated with too few patients per cohort. We identified significantly reduced survival in a total of five cancers types that expressed high levels of one of these three genes (Fig. 5b).

### Sex disparity of p53-STRING gene expression in normal adults

We next examined the relative gene expression levels between males and females of the X-linked p53-STRING set in tissues classified as normal and discriminated as cancer-free, from the TCGA cohorts across all the disparity cancers. While these are referred to as matched normals, we note that these may be affected by the existence of cancer cells and/or by adjacent tumour tissues in these patients (Supplementary Data 5, TAB2). In the matched normals, *KDM6A*, *DDX3X*  and *UBA1* were significantly differentially expressed among the p53-STRING set. For comparison, we extended this test to the healthy samples of the 1000 genomes project, which are lymphoid in origin. Among these, statistically significant differential expression (DE) was identified between the sexes for 13 of the p53-STRING set genes (among the 95 X -linked DE genes with adjusted *p*-value ≤ 0.05, Limma, Benjamini–Hochberg adjustment). All 13 genes were expressed at higher levels in females than in males (Supplementary Fig. 5; Supplementary Data 5, TAB3) with *KDM6A*, *DDX3X*  and *UBA1* prominent, which complements our findings in the normals matched to the tumours.

## Discussion

The well-documented, higher incidence of lethal, non-reproductive cancers in males than females is attracting a growing interest into its underlying causes (eg., refs. ^2,3^), however, the picture is far from clear. Guided by our previous study demonstrating sex disparity in a compound mutant p53 mouse model^8^, together with the unprecedented incidence of *TP53* mutations in human cancers, we tested the contribution of p53 to cancer sex disparity. We identified three levels of connection between p53 and sex disparity in somatic non-reproductive cancers. For the first time, our statistical analyses predict a significantly higher frequency of cancers with mutated *TP53* in males than females, for 12 common sporadic cancers, across the US population (as determined through the integration of TCGA and SEER data). The exception is KIRC, which was more common in males than females, but with exceptionally few *TP53* mutations. Notably, in KIRC, although *TP53* mutation is infrequent, poorest survival corresponds with high levels of MDM2, which emphasises the importance of evading p53 tumour suppression also in this disease^48^. This outlier dismisses a direct correlation between cancer prevalence and *TP53* mutation as its explanation. These findings are in line with somatic *TP53* mutations accumulating preferentially in tissues arising from the embryonic layers of the ectoderm and the endoderm, in contrast to germline *TP53* mutations which at least for non-reproductive cancers, preferentially occur in tissues derived from the mesoderm^23^, which includes the kidney.

It is pertinent to our study that the ethnic population composition of TCGA and SEER correlate remarkably closely. The verified TCGA^49^: SEER^21^ proportions are for Whites ~73%: ~78%, respectively, for Black or African Americans ~8%: 13%, respectively; and are near equivalent for Asians. These very similar proportions are relevant as *TP53* mutation frequencies appear lower in Caucasians for certain cancer types (reviewed in ref. ^49^). It is also relevant to note that TCGA is comprised of primary disease, regardless of whether originating from resections or biopsies, sampled from metropolitan centers. SKCM is the only exception, but most of these tumours are wt *TP53*^50^. The nature of these samples may have contributed to an underestimate of *TP53* mutation incidence in the population, as metastatic disease is frequently associated with mutation of this tumour suppressor (reviewed in ref. ^51^) and non-metropolitan disease is frequently more severe^52^. In sum, the incidence of *TP53* mutation in the US population may be even higher than our calculations predict, with males at greater risk than females.

Our findings indicate that tissue-specific vulnerability to somatic *TP53* mutation is exacerbated by patient sex. This is in line with the specific instance of glioblastoma astrocytes, in which components of the p53 and RB pathways were recently reported to contribute to sex disparity^53,54^. This could result from a number of causes. One possibility is that male non-reproductive tissues (pre-cancer) may be more exposed to carcinogens and/or prone to spontaneous pathogenic *TP53* mutation than their female counterparts. Alternatively, females may have a better tumour-suppressive capacity due to a greater ability to clear or restrain cells with *TP53* mutations, compared with males. Testing these hypotheses will require prospective sampling for *TP53* mutations in large data sets from healthy populations, across multiple tissues, with temporal follow-up. Currently, this information is unavailable. Significantly, among the disparity cancers, the occurrence of *TP53* mutation corresponded with the worst survival outcomes. This suggests that *TP53* mutation is an overriding oncogenic event. Indeed, many of the regulatory and auto-regulatory loops with p53 are operative only in the context of wt p53^55^. As males have higher cancer incidence and as we demonstrate, a greater frequency of *TP53* mutations than females, this is a likely contributor to their poor cancer survival statistics. While this study is restricted to the US population, similar statistics would be expected across the western world.

The second major level of contribution of p53 to sex disparity is through its connection to the X allosome. While females have two chromosomes, developmental Xi reduces expression close to that of the single X chromosome in males. Females are protected from germline mutations by Xi and mosaicism (reviewed in ref. ^56^). Accordingly, X-linked inherited diseases manifest predominantly in males, frequently at a young age. Notably, with our colleagues we recently identified that p53 drives Xi in females during development^57^, and its absence in females is fatal, largely associated with neural tube defects^58^. In contrast, most cancers arise from somatic mutations in the aging population, where we predicted alternative mechanisms must account for lower cancer incidence in females. We uncovered a novel p53-X-chromosome network (Supplementary Data 1), with cancer protection potential that is particularly advantageous for females. Generally in cancers, we identified that somatic X-linked gene mutations, including the genes of the p53-STRING set, occur twice as frequently in females as in males, reflecting their distinct X-chromosome copy numbers. However, this does not correlate with cancer incidence. In contrast, we identified that our newly identified X-linked p53-STRING gene set are at peculiar risk of deregulation in male cancers.

The functional relevance of this p53-STRING set emerged though studying the mRNA levels of these genes. Expression analysis (by GSEA) of the 11 p53-STRING set genes reported to negatively regulate p53, exposed a higher level of expression in cancers with wt *TP53*, than in the matched normal tissues (Fig. 5a). This proved to be statistically significant in seven cancers in males and six in females, although for all cancers this trend was evident. Survival analysis examining high expression levels of three of these genes (*UBE2A*, *MAGEA2* and *UTP14A*) corresponded with poorer survival in five different cancers (Fig. 5b). These findings align with compromised wt p53 activity, through enhanced levels of its negative regulators posing a major cancer risk.

As each cancer develops along a different trajectory of mutations^59^, we did not expect that all cancers would be impacted equally by these genes. It is expected that in different cancer types and for each individual cancer case, different combinations of genes will be involved, with different permutations of deregulated expression and/or mutations. A particular permutation would be influenced by the status of *TP53*, whether these X-linked genes are either positive or negative regulators/effectors, and further whether these genes themselves have undergone mutation.

An interesting translational angle arises from this data. Wt *TP53* status is generally a good prognostic marker for response to conventional genotoxic drugs due to their reliance on wt p53 activation (reviewed in ref. ^60^). In the context of high expression of negative regulators of wt *TP53*, as exposed in our study, p53 activation is likely to be ineffective, leading to poor response, and consequently to poor survival. Our findings suggest clinical pertinence, where altered protein expression of negative p53 regulators such as these offer potential value as prognostic markers (as suggested for UBE2A for liver cancer^61^ and UTP14 for colorectal cancer^62^ and MAGEA2 for lung cancer^63^). Also when overexpressed, these genes represent targets for therapy in these male dominated cancers. A specific example is HDACi trichostatin A plus etoposide administered to treat cancers with elevated MAGEA2^47^. Our study lays a foundation for developing rational strategies to treat cancer patients on the basis of expression levels of these regulators in the context of *TP53* mutational status. The greater prevalence of these cancers in males, predicts disproportionate application of these tools for prognosis and treatments between the sexes.

In contrast to these cancer risks, in females, both in the cancer-matched normal TCGA samples and the 1000 genomes project, we identified elevated expression of a number of p53-STRING set genes that are noted for tumour-suppressive capacity. Their higher levels in females are predicted to afford them a more robust innate cancer protection compared with males, who were identified with inherently lower expression. The most prominent of these are candidate tumour suppressors; KDM6A (lysine demethylase 6 A), DDX3X  (dead-box RNA helicase 3) and UBA1. Notably, KDM6A inhibits EMT by antagonising TGF-β induced genes, which are instrumental in this process^64^. At the other extreme, elimination of KDM6A is proposed as an independent marker of prognosis for pancreatic cancer^65^. DDX3X protects against replication stress through its regulation of DNA damage repair^66^. Pertinently, a peculiar male vulnerability to DDX3X mutation has just been reported for melanoma^67^, a disease where *TP53* is predominantly wt. In sum, the cancer protective potential of KDM6A and DDX3X in normal females preferentially, is emphasised by our data, which also reinforces their particular mutation risk in male cancers. UBA1 is also essential for proper response to replication stress and DNA damage^68^. Specifically, UBA1 localises to DNA breaks and mediates ATR/Chk1 signalling^69^. A vital ancestral role for Uba1 is supported by its highly conserved counterpart in Drosophila, which is implicated as a tumour-suppressor gene. Apoptosis is defective in the absence of Uba1 in Drosophila^70^, consistent with weak alleles of Uba1 protecting from cell death and strong Uba1 alleles driving cell cycle arrest^71^.

Our third novel link of p53 to sex disparity entails the unique protection of females from expression of mutations from a set of X-linked genes connected to its function. In marked contrast to the males, most X-chromosome exome mutations in the female cancers were not detected at the mRNA level. This phenomena is distinct from the X-chromosome loss identified in solid tumours with aneuploidy^72^. The exceptional abundance of female NEMs among the p53-STRING gene set, across the 12 disparity cancers, suggests that this phenomenon is not simply random (Fig. 4). The p53-STRING genes that exhibit NEMs, encode proteins that occur in pathways that are instrumental in preserving genomic integrity, through epigenetic regulation (dominated by histone modification: H3-K9 and ubiquitination) and telomere maintenance (Supplementary Fig. 3b). We argue that the prevalence of NEMs among the genes of the p53-STRING set in females reflects an important level of resistance to cancer development that is not afforded to males. Among these NEMs, specific genes stood out as highly significant and pertinently, their encoded proteins have functional links to p53 activation pathways. These include FLNA, a p53-regulated^73^, cytoskeleton remodeler, that can either suppress or promote tumours depending on its cleavage and cellular location  (reviewed in ref. ^74^). MED1*2*, a key component of the pre-initiation complex that promotes *p21* transcription^75^; HUWE1, an E3 ligase of p53 that regulates DNA damage response to UV damage^76^; and ATRX, that works with p53 to mediate DNA repair^77^. Breaching these defence barriers in females during cancer onset is predicted from the identification of expressed mutations among the p53-STRING set in cancer samples. Intriguingly, GO analysis of cancer EMs, including many of the p53-STRING genes, exposed distinct pathway disruption between females and males.

Our studies discovered three levels of connection between p53 and cancer sex disparity. First, the frequency of males with *TP53* mutation is greater than females, across 12 disparity cancers in the US population. Poorer survival outcomes for patients with mutant *TP53* cancers is consistent with *TP53* status contributing to cancer sex disparity. Second, we uncovered a set of X-linked genes encoding proteins that interact with p53. Due to the distinct mode of X-chromosome expression between the sexes, males are peculiarly vulnerable to the consequences of X-linked gene alterations compared with females. These two levels are overlaid by a third, exclusively female barrier that restricts expression of X-linked somatic gene mutations, particularly for the p53-STRING set. We propose that the combination of all three levels amplify the impact of p53 on cancer sex disparity and that disruption of this network defines a disproportionate male cancer risk.

Patient sex is currently not a major dictator of therapeutic choices. Our findings expose differential molecular sensitivities between the sexes in cancers. They offer scope for explaining sex discrepancy in treatment efficacy. Importantly, we predict that male and female cancers are likely to benefit from distinct treatment options, cancer surveillance and prevention. While our study focuses on cancer, it exposes a potential defence phenomenon for females against other sporadic diseases and syndromes caused by X-chromosome mutations.

## Methods

### Data source and propensity scores

TCGA^22^ patient data were accessed and processed for analysis guided by the approach of Yuan et al.^2^. Briefly, for proper statistical analysis, TCGA cohorts must be appropriately adjusted for cofounding factors. This was achieved using a re-weighting system based on propensity scores calculated using the algorithm developed by Rosenbaum and Rubin^78^ and adapted by Yuan et al.^2^. Specifically, TCGA clinical data (e.g., patient sex, age at diagnosis, tumour stage, race and smoking status) for the 12 cancer types were obtained from The Broad Institute GDAC Firehose^79^. Initially, propensity scores based on patient sex were calculated using logistic regression. Then, using these scores, a matching weight scheme was performed to re-weight the samples^80^. This scheme was adopted to balance the propensity scores and in turn the covariates. A strict checking loop was implemented throughout this process to perpetuate continuous revision until all covariates were balanced between males and females. The weights calculated by the propensity scores algorithm were used in a number of statistical models to compare and assess significance for the different molecular data (non-expressed mutations [NEMs], expressed mutations [EMs], mRNA differential expression) using patient sex as the independent variable.

### Survival analysis

In order to assess the prognostic value of the expression of the p53-STRING set genes among the disparity cancers, normalised gene expression and survival data from the TCGA data set were accessed using The Broad Institute GDAC Firehose^79^ and analysed in R. Kaplan–Meier survival curves were generated using the top and bottom medians of gene expression, and differences in survival distributions between medians were tested using a log-rank test.

### Exome mutations, RMAF and mutation classification

For these non-reproductive cancers, a full list of exome level somatic mutations was downloaded from Genomics Data Commons Data Portal and GDAC Firehose, more precisely, the Oncotator^81^; annotated mutations were analysed. Mutations classified as “3’UTR”, “5’UTR”, “Frame_Shift_Del”, “Frame_Shift_Ins”, “Missense_Mutation”, “Nonsense_Mutation”, “Splice_Region” and “Splice_Site” were used to focus on mutations with the potential of having a more profound functional impact. Patients with available sex annotation and <15,000 somatic mutations were analysed.

To assess whether DNA mutations in the X chromosome were transcribed into RNA, accounting for Xi, we classified mutations into two kinds: NEMs and EMs, and tested differences in numbers between males and females. For each cancer type, we combined the exome somatic mutation list with the corresponding TCGA RNASeq BAM files using the Cancer Genomics Cloud platform by Seven Bridges (http://www.cancergenomicscloud.org/). For each X-chromosome DNA mutation, we defined a new metric: the RNA-mutated allele frequency (RMAF), an equivalent to variant allele frequency (VAF) commonly used to call mutations at DNA level (Supplementary Figs. 6, 7). RMAF was calculated by piling all overlapping mRNA reads at the genomic position, where the DNA mutation was called. When a sample had multiple somatic DNA mutations present in the same gene, only the mutation with the highest RMAF was used. When mRNA reads were not available or too few (<10) at the genomic position for a mutation, we calculated RMAF the following way: firstly, we checked if the gene in question was generally expressed in the corresponding cohort (male or female; with at least five counts per million (CPM) in the majority of samples), if no expression was found, the mutation was discarded. Secondly, we calculated a z-score based on the gene expression of the corresponding sample relative to its corresponding cohort (male or female). If the expression of the gene was substantially diminished (i.e., 4 standard deviations below the mean; z-score ≤ −4) we concluded that the mutation had a functional effect in that sample (a knockdown effect) and assigned a RMAF of 1. Otherwise (z-score > -4), we discarded the mutation as its impact cannot be assessed. When mRNA reads were sufficiently abundant, RMAFs were calculated using Python and the Pysam library (https://github.com/pysam-developers/pysam), a wrapper of htslib and samtools^82^. Based upon the relative RMAF distributions in the male and female cancer samples, we established two thresholds in an effort to account for tumour purity and heterogeneity and classified mutations with an RMAF greater or equal to the threshold of 0.75 as EMs and those less or equal to 0.2 as NEMs.

### NEMs, EMs and the X chromosome

After quantifying NEMs and EMs, we applied the propensity score algorithm and tested for significant differences in NEMs and EMs numbers between male and female patients. We applied a weighted chi-squared test and highlighted genes that had adjusted *p*-values ≤ 0.05 (Benjamini–Hochberg adjustment). We calculated a statistic for each of the 561 genes with NEMs, using the propensity score weightings which, under the null hypothesis: no association between a patients’ sex (female vs male) and no association with the status of the expression of the specific gene mutation (expressed versus not) would be asymptotically distributed as chi-squared on one degree of freedom. By combining these statistics across all the genes and test for differences in NEMs numbers between the p53-STRING set and other X-chromosome genes, we collected the associated chi-scores: the signed square roots of the chi-squared statistics, with the sign determined in the same way for all genes. We assigned a plus sign if the proportion of NEM in females was larger than the proportion of NEM in males; and the converse was discriminated with a minus sign. These chi-scores should be asymptotically distributed as standard normal (mean 0, standard deviation 1) under the null hypotheses, for both the p53-STRING set and other genes. We tested (Wilcoxon rank test) the difference in distribution between these two groups and found that p53-STRING set genes host significantly larger number of NEMs compared with other genes in the X chromosome.

### Differential expression

Raw gene-level counts for each cancer type were downloaded from The Broad Institute GDAC Firehose^79^. Reads were converted to counts per million (CPM). Lowly expressed genes (fewer than 5 CPMs) were discarded. CPM counts were adjusted and normalised by library size using the R package edgeR^83^. By accessing the clinical data and mutation lists, two groups were analysed: Wt-*TP53* Tumours and Mt-*TP53* tumours (all samples with pathogenic *TP53* mutations), each one stratified by patient sex. In addition, RNAseq count data from 465 healthy lymphoblastoid cell lines (derived from normal bloods) was downloaded from the 1000 Genomes project (https://www.ebi.ac.uk/arrayexpress/experiments/E-GEUV-1)^84^. Normalisation was also performed using edgeR^83^. By accessing the available clinical data two groups were analysed: males and females. Differences in gene expression between these groups were computed with the R package Limma^85^, and significant changes were inferred by obtaining *p*-values and by adjusting for multiple hypothesis testing (Benjamini–Hochberg adjustment), false discovery rates were calculated. Propensity scores were also considered in the differential expression analysis.

### Gene set enrichment analysis

To further investigate the role of p53-STRING genes in the disparity set, after performing differential expression analysis (normal versus wt *TP53* tumours), the corresponding gene lists ranked by fold change were analysed for enrichment of negative X-linked p53 regulators using the fgsea package in R^86^. Normalised enrichment scores (NES) and adjusted *p*-values (adjustment by FDR) were used to highlight the enrichment of these genes in wt-p53 tumours.

### ClueGO cytoscape analysis

Gene Ontology (GO) analysis of the gene sets was performed in ClueGO Cytoscape. The following parameters were selected: GO tree interval between levels 3 and 8; GO terms with at least three proteins and 4% of proteins; a kappa score of 0.4 and only GO terms with a *p*-value ≤ 0.05 were selected.

### STRING analysis

Search Tool for the Retrieval of Interacting Genes/Proteins (STRING) database tool^31^ was adopted for analysis of X-linked genes that link to *TP53* (version 10.5; https://version-10-5.string-db.org/cgi/network.pl?taskId=PpCASJpZQ8Du). *TP53* was coincidently included in the gene input, to define association. Parameters of selection included: textmining, experiments co-expression, gene-fusion, and co-occurrence. We ignored genes that sorted into a confidence score <0.3.

## Supplementary information

Supplementary Information

Description of Additional Supplementary Files

Supplementary Data 1

Supplementary Data 2

Supplementary Data 3

Supplementary Data 4

Supplementary Data 5

## Data Availability

The data generated and analysed during this study are included in this published article and its supplementary information files.

## Code availability

Code for normalisation, differential expression, log ratios tests, survival analysis, propensity scores calculation and RMAF calculation is available at https://github.com/fcaramia.
