# Supplementary material for: Identification of cancer sex-disparity in the functional integrity of p53 and its X chromosome network
Source: Nat Commun. 2019 Nov 26;10:5385. doi: 10.1038/s41467-019-13266-3 (PMC6879765; doi:10.1038/s41467-019-13266-3)
Supplement: Supplementary file 1 — Supplementary Information [file 41467_2019_13266_MOESM1_ESM.pdf]

Haupt et al., Supplementary Figures and Supplementary Table 1

Identification of cancer sex-disparity in the functional integrity of p53 and its X chromosome network

Supplementary Table 1: Application of the laws of probability to infer the rate of male and female *TP53* mutation frequency in cancers in the general population: STAD the most common male cancer as an example

Notation. Let $pr(A|B)$ denote the probability or relative frequency of the event A, within some well defined population, given an event or condition B. Here the vertical bar | is read as "given". For example, if μ denotes the event that a person in a population has a pathogenic *TP53* mutation, and M denotes the event that a person is male, then $pr(\mu|M)$ is the probability that a male person in that population has a pathogenic *TP53* mutation (where 80% of *TP53* mutations are predicted to be pathogenic[1]). In general this will depend on the age and other characteristics of members of the population, but as what follows is simply an illustrative analysis, we will only consider the sex of the person. Let F denote the event that a person is female, C that a person has a cancer of one of the kind under discussion, and nC that a person does not have a cancer of the kind under discussion.

Our interest lies in comparing $pr(\mu|M)$ with $pr(\mu|F)$, in the knowledge that there is no information on the population frequencies of pathogenic mutations of *TP53*, outside the context of cancer. One further piece of notation is the ampersand &, simply read as "and". For example, $pr(\mu|M\&C)$ is the probability that a male with cancer has a pathogenic mutation in *TP53*: "$|M\&C$" being read "given the person is male and has a cancer.

We will now start with certain established facts, and draw some tentative conclusions using STAD as an example.

Fact 1. TCGA data shows that for STAD*: $pr(\mu|M\&C) \approx pr(\mu|F\&C) \approx 0.5.$

Fact 2. SEER data** shows that: $pr(C|M) \approx 2 \times pr(C|F) \approx 0.00010.$

We will now use these facts, the laws of probability and a plausible assumption to infer that $pr(\mu|M) > pr(\mu|F)$, perhaps even that $pr(\mu|M) \approx 2 \times pr(\mu|F)$.

Derivation. A male or female with a pathogenic *TP53* mutation either has a cancer or the type under discussion, or they do not. These events are mutually exclusive, and so the probabilities add:

(1) $pr(\mu|M) = pr(\mu\&C|M) + pr(\mu\&nC|M),$
(2) $pr(\mu|F) = pr(\mu\&C|F) + pr(\mu\&nC|F).$

Next we use one of the basic laws of probability, here stating that $pr(\mu\&C|M) = pr(C|M) \times pr(\mu|C\&M),$ and similarly for the other 3 terms. In other words,

(3) $pr(\mu|M) = pr(C|M) \times pr(\mu|C\&M) + pr(nC|M) \times pr(\mu|nC\&M),$
(4) $pr(\mu|F) = pr(C|F) \times pr(\mu|C\&F) + pr(nC|F) \times pr(\mu|nC\&F).$

We can now make use of Facts 1 and 2, substituting which gives

(5) $pr(\mu|M) \approx 0.00010 \times 0.4964 + (1-0.00010) \times pr(\mu|nC\&M),$
(6) $pr(\mu|F) \approx 0.00005 \times 0.4713 + (1-0.00005) \times pr(\mu|nC\&F).$

We now examine what is not known in these last two equations: *pr(μ|nC&M),* and the corresponding quantity for females *pr(μ|nC&F).* These are the relative frequencies of pathogenic mutations among people, either male or female, without cancer.

Plausible assumption: *pr(μ|nC&M)* and *pr(μ|nC&F)* are both quite small in comparison with the frequencies of cancer in males and females.

This is almost a consequence of the definition of a pathogenic mutation.

Conclusion: Given Facts 1 and 2, and the Plausible Assumption, and rounding a little

$pr(\mu|M) \approx 0.00010\times 0.4964 = 0.000050,$
$pr(\mu|F) \approx 0.00005\times 0.4713 = 0.000025.$

This predicts that in the general population, the incidence of pathogenic TP53 mutations in male STAD, the most common non-reproductive cancer in males is ~two times as frequent as in female STAD.

Similarly, the probability for 10 additional non-reproductive cancers have been calculated and the TP53 mutation incidence in the general population is predicted to be higher in cancers of males, compared with females for all the 11 cancers of the 'disparity-set' (Table 2).

Key:
* From Table 1. Broad Institute source of TCGA STAD data
Females: 74 mutant *TP53*/157 cancers (47.13%)
Males: 139 mutant *TP53*/280 cancers (49.64%)

** From SEER data
Females: 5.3 STAD cases/100,000 General Population (0.00005)
Males: 10.1 STAD cases/100,000 General Population (0.00010)

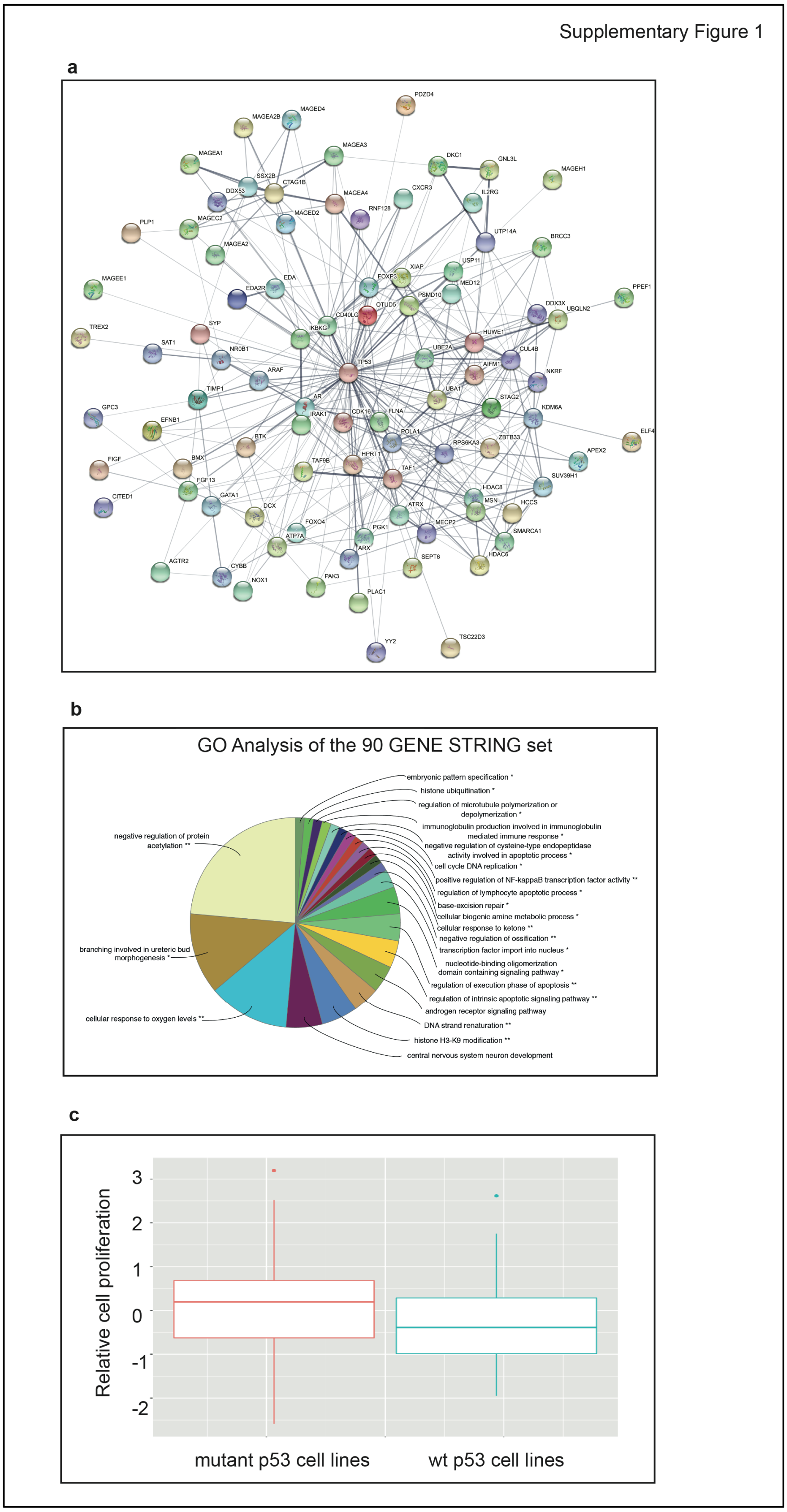

Supplementary Figure 1. p53-STRING set and functional linkages. (a) X-linked genes that link to p53 in the STRING database as defined using the parameters of: Textmining; Experiments; Co-

expression ; Genefusion, Co-occurrence and a confidence of >0.3. (**b**) GO Analysis of the 90 genes of the p53-STRING set, analysed with the inclusion of *TP53*. (c) Achilles Project data of relative proliferation for 135 cell lines subjected to HUWE1 siRNA and stratified for *TP53* status. Statistical significance was analysed by a t-Test; p-value=0.05.

Supplementary Figure 2

a

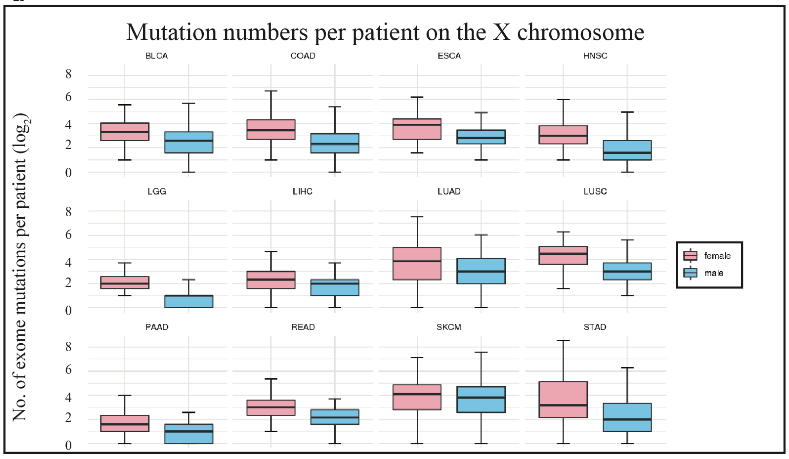

DATASET	p-value
BLCA	5.99E-58
COAD	2.92E-234
ESCA	5.28E-34
HNSC	7.83E-160
LGG	6.73E-41
LIHC	7.15E-10
LUAD	8.56E-155
LUSC	0
PAAD	2.14E-57
READ	3.65E-14
SKCM	2.88E-21
STAD	6.59E-299

b

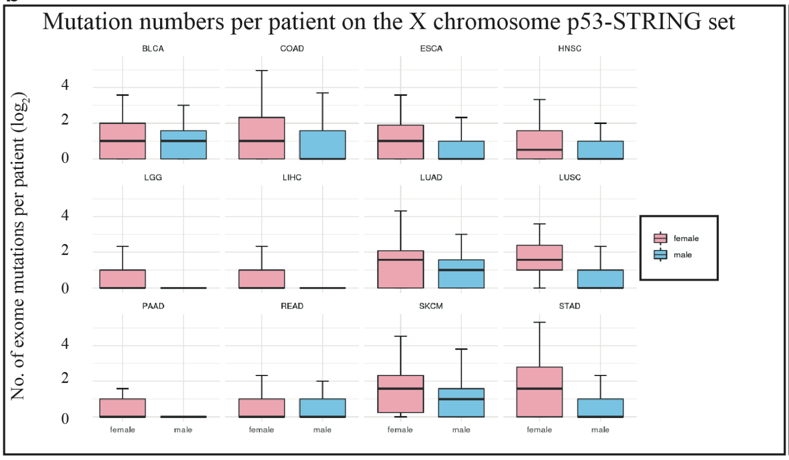

DATASET	p-value
BLCA	4.93E-05
COAD	1.99E-09
ESCA	0.000854911
HNSC	1.81E-09
LGG	0.132993147
LIHC	0.104870839
LUAD	2.07691E-12
LUSC	1.04E-26
PAAD	5.83E-05
READ	0.738893609
SKCM	9.07E-06
STAD	1.89E-27

c

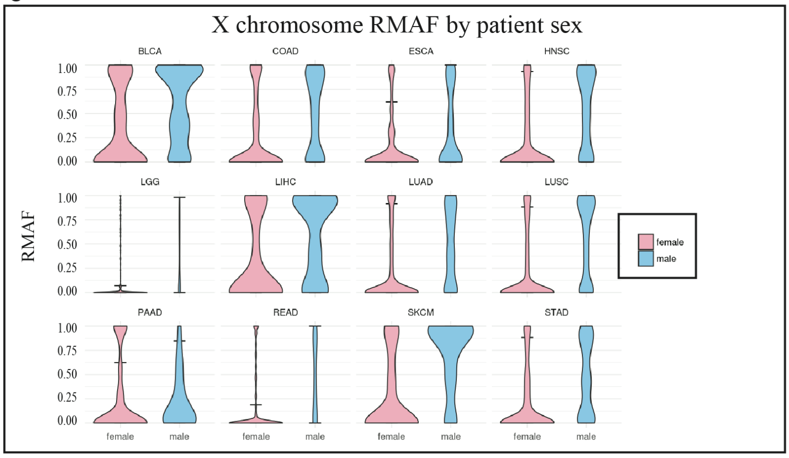

DATASET	p-value
BLCA	3.40E-25
COAD	3.19E-41
ESCA	1.60E-07
HNSC	9.43E-49
LGG	1.56E-13
LIHC	2.22E-09
LUAD	1.66E-63
LUSC	1.05E-88
PAAD	0.992728412
READ	3.03E-11
SKCM	1.54E-19
STAD	5.72E-41

Supplementary Figure 2. Frequency of X chromosome mutation was greater in females with corresponding less RMAF than males, for the 12 disparity cancers. Plotted for each cancer individually according to patient sex are (**a**) the number of exome mutations per patient for all the X chromosome genes; (**b**) the number of exome mutations per patient for

the p53-STRING set of genes specifically; and (**c**) RMAF. Females are indicated in pink and males in blue.

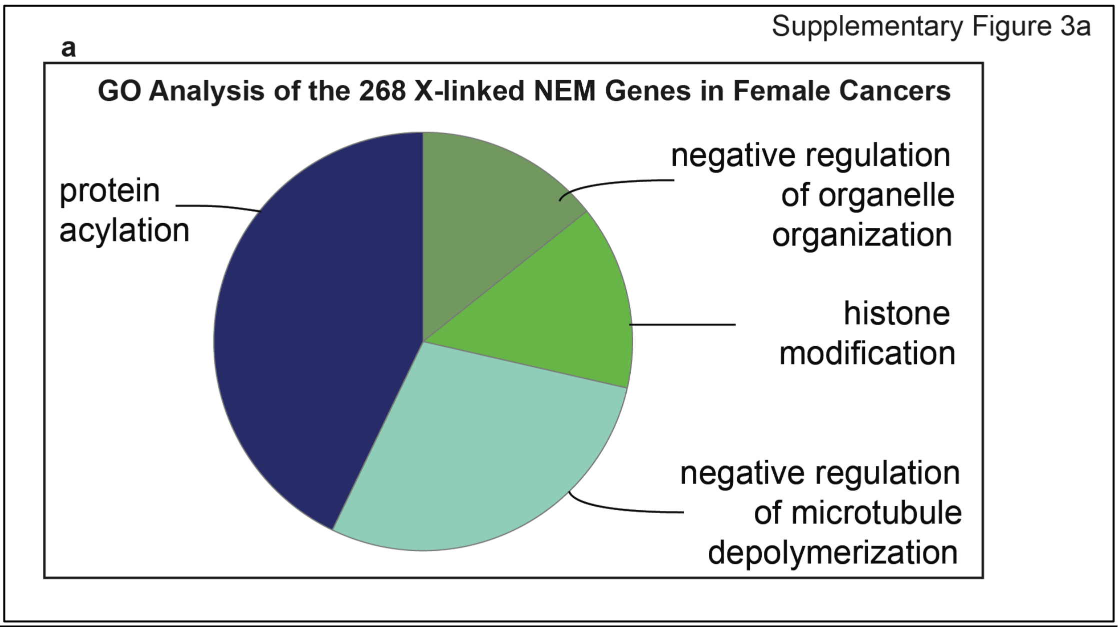

Biological Processes	Associated Genes Found	% Associated Genes	GOID
negative regulation of microtubule depolymerization	[FGF13, HDAC6, MID1, MID1IP1]	18	GO:0007026
regulation of microtubule depolymerization	[FGF13, HDAC6, MID1, MID1IP1]	17	GO:0031114
internal protein amino acid acetylation	[HCFC1, HDAC8, JADE3, MECP2, MORF4L2, MSL3, NAA10, OGT, TAF1, TAF9B]	6	GO:0006475
histone acetylation	[HCFC1, HDAC8, JADE3, MECP2, MORF4L2, MSL3, OGT, TAF1, TAF9B]	6	GO:0016573
protein acylation	[HCFC1, HDAC8, JADE3, MECP2, MORF4L2, MSL3, NAA10, OGT, PORCN, TAF1, TAF9B, ZDHHC9]	5	GO:0043543
histone modification	[ATRX, BCOR, CUL4B, FMR1, HCFC1, HDAC6, HDAC8, HUWE1, JADE3, KDM5C, MECP2, MORF4L2, MSL3, OGT, PHF8, SUV39H1, TAF1, TAF9B, TBL1X, UBE2A]	4	GO:0016570
negative regulation of organelle	[ATRX, BCOR, FGF13, GNL3L, HDAC6, HDAC8, MECP2, MED12, MID1,	4	GO:0010639

organization	MID1IP1, MTM1, NAA10, PHF8, PSMD10, WAS]		

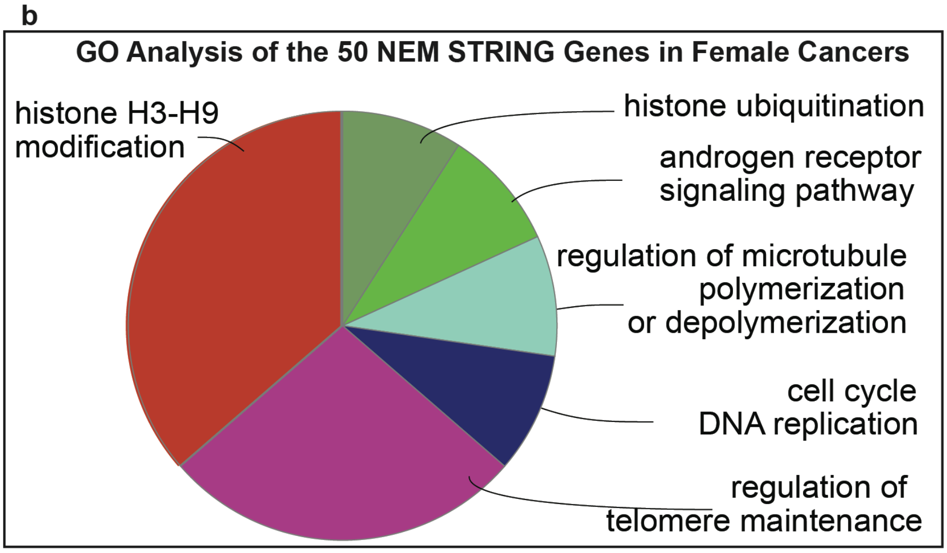

Biological Processes	Associated Genes Found	% Associated Genes	GOID
histone H3-K9 trimethylation	[ATRX, MECP2, SUV39H1]	21	GO:0036124
protein localization to chromosome, telomeric region	[ATRX, DKC1, GNL3L]	10	GO:0070198
histone H3-K9 methylation	[ATRX, MECP2, SUV39H1]	10	GO:0051567
histone H3-K9 modification	[ATRX, HDAC8, MECP2, SUV39H1]	10	GO:0061647
peptidyl-lysine trimethylation	[ATRX, MECP2, SUV39H1]	8	GO:0018023
histone ubiquitination	[CUL4B, HUWE1, UBE2A]	7	GO:0016574
protein localization to chromosome	[ATRX, DKC1, GNL3L, HDAC8]	5	GO:0034502
cell cycle DNA replication	[ATRX, POLA1, STAG2]	5	GO:0044786
regulation of telomere maintenance	[ATRX, DKC1, GNL3L, HDAC8]	5	GO:0032204
regulation of microtubule polymerization or depolymerization	[FGF13, HDAC6, MECP2]	4	GO:0031110

androgen receptor signaling pathway	[HDAC6, MED12, TAF1]	4	GO:0030521

Supplementary Figure 3. GO analysis of X-linked Non-Expressed Mutations (NEMs) for all Female disparity cancers. (a) GO analysis of the pathways enriched for the genes with comprising the 268 significant NEMs, as a VENN diagram; and listing of enriched pathways and gene identities. (**b**) GO analysis of the 50 significant p53-STRING gene set as a VENN diagram; and listing of enriched pathways and gene identities

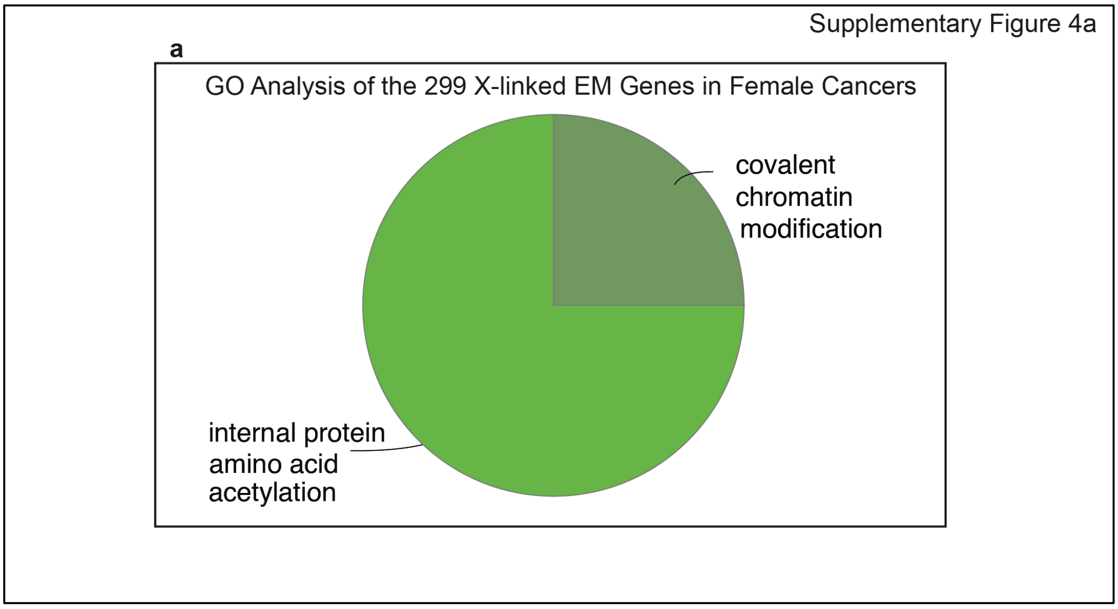

Biological Processes	Associated Genes Found	% Associated Genes	GO.ID
internal protein amino acid acetylation	[HCFC1, HDAC8, JADE3, MECP2, MORF4L2, MSL3, NAA10, NAP1L2, OGT, TAF1, TAF9B]	7	GO:0006475
histone acetylation	[HCFC1, HDAC8, JADE3, MECP2, MORF4L2, MSL3, NAP1L2, OGT, TAF1, TAF9B]	7	GO:0016573
peptidyl-lysine acetylation	[HCFC1, HDAC8, JADE3, MECP2, MORF4L2, MSL3, NAP1L2, OGT, TAF1, TAF9B]	6	GO:0018394
protein acetylation	[HCFC1, HDAC8, JADE3, MECP2, MORF4L2, MSL3, NAA10, NAP1L2, OGT, TAF1, TAF9B]	6	GO:0006473
protein acylation	[HCFC1, HDAC8, JADE3, MECP2, MORF4L2, MSL3, NAA10, NAP1L2, OGT, PORCN, TAF1, TAF9B, ZDHHC9]	6	GO:0043543
histone modification	[ATRX, BCOR, CUL4B, FMR1, HCFC1, HDAC6, HDAC8, HUWE1, JADE3, KDM5C, KDM6A, MECP2,	5	GO:0016570

	MORF4L2, MSL3, NAP1L2, OGT, PHF8, RBBP7, SUV39H1, TAF1, TAF9B, TBL1X, UBE2A]		
covalent chromatin modification	[ATRX, BCOR, BCORL1, CUL4B, FMR1, HCFC1, HDAC6, HDAC8, HMGN5, HUWE1, JADE3, KDM5C, KDM6A, MECP2, MORF4L2, MSL3, NAP1L2, OGT, PHF8, RBBP7, SMARCA1, SUV39H1, TAF1, TAF9B, TBL1X, TSPYL2, UBE2A]	5	GO:0016569
peptidyl-lysine modification	[ATP7A, ATRX, BCOR, GNL3L, HCFC1, HDAC6, HDAC8, JADE3, KDM6A, MECP2, MORF4L2, MSL3, NAP1L2, OGT, SUV39H1, TAF1, TAF9B, ZBED1]	5	GO:0018205

Supplementary Figure 4b

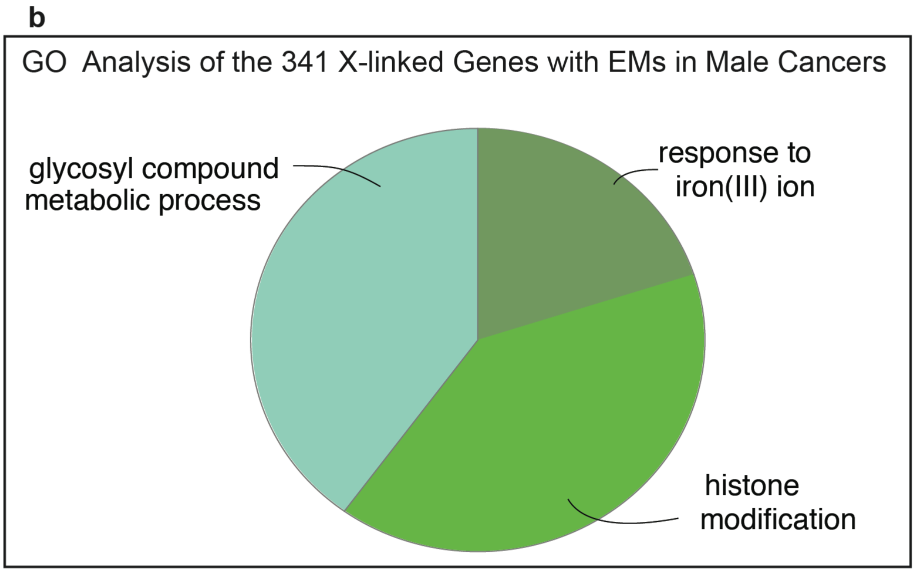

**Biological Processes**	**Associated Genes Found**	**% Associated Genes**	**GO.ID**
response to iron(III) ion	[ATP7A, G6PD, UBL4A]	50	GO:0010041
nucleoside metabolic process	[CASK, CTPS2, DLG3, HPRT1, MED12, MPP1, PRPS1, PRPS2, PUDP, UPRT]	7	GO:0009116
glycosyl compound metabolic process	[CASK, CTPS2, DLG3, GLA, HPRT1, MED12, MPP1, PRPS1, PRPS2, PUDP, UPRT]	7	GO:1901657
histone	[ATRX, BCOR, BRCC3, CUL4B,	5	GO:0016570

modification	FMR1, HCFC1, HDAC6, HDAC8, HUWE1, JADE3, KDM5C, KDM6A, MECP2, MORF4L2, MSL3, OGT, PHF8, RBBP7, SUV39H1, TAF1, TAF9B, TBL1X, UBE2A]		
covalent chromatin modification	[ATRX, BCOR, BCORL1, BRCC3, CUL4B, FMR1, HCFC1, HDAC6, HDAC8, HUWE1, JADE3, KDM5C, KDM6A, MECP2, MORF4L2, MSL3, OGT, PHF8, RBBP7, SMARCA1, SUV39H1, TAF1, TAF9B, TBL1X, TSPYL2, UBE2A]	4	GO:0016569

Supplementary Figure 4. GO analysis of X-linked Genes with Expressed Mutations (EMs) for all disparity cancers. (a) GO analysis of the pathways enriched for the genes containing the 299 EMs in female cancers, as a VENN diagram; and listing of enriched pathways and gene identities. (**b**) GO analysis of the 341 genes with EMs in the p53-STRING gene set as a VENN diagram; and listing of enriched pathways and gene identities.

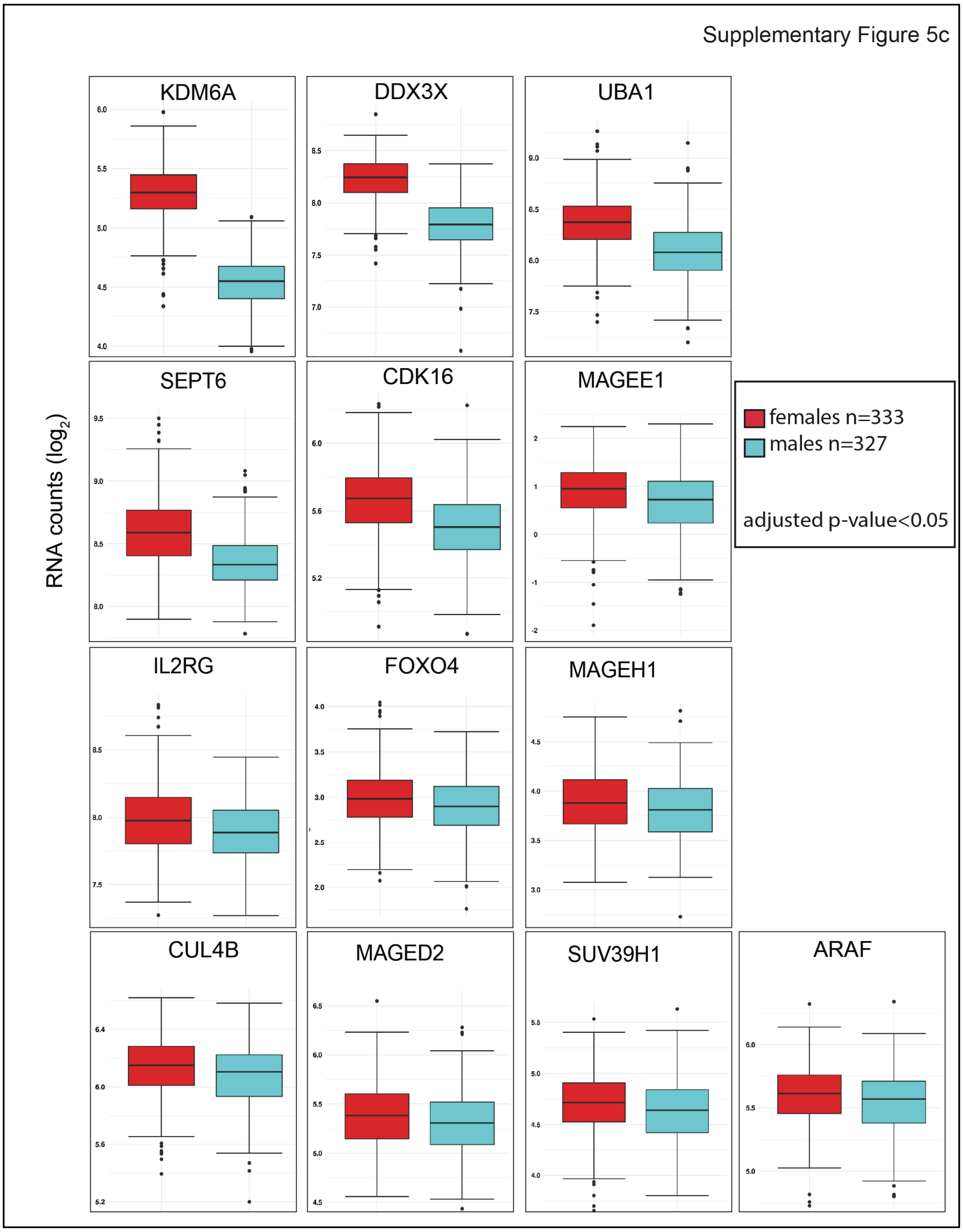

Supplementary Figure 5. Expression of 13 p53-STRING genes was greater in normal females than males as identified in blood-derived lymphoblastoid cell lines of the 1000 genomes project. Differential expression (DE) analysis of all expressed X-linked genes identified multiple p53-STRING genes to be significantly more expressed in healthy females than males (adjusted p-value <0.05).

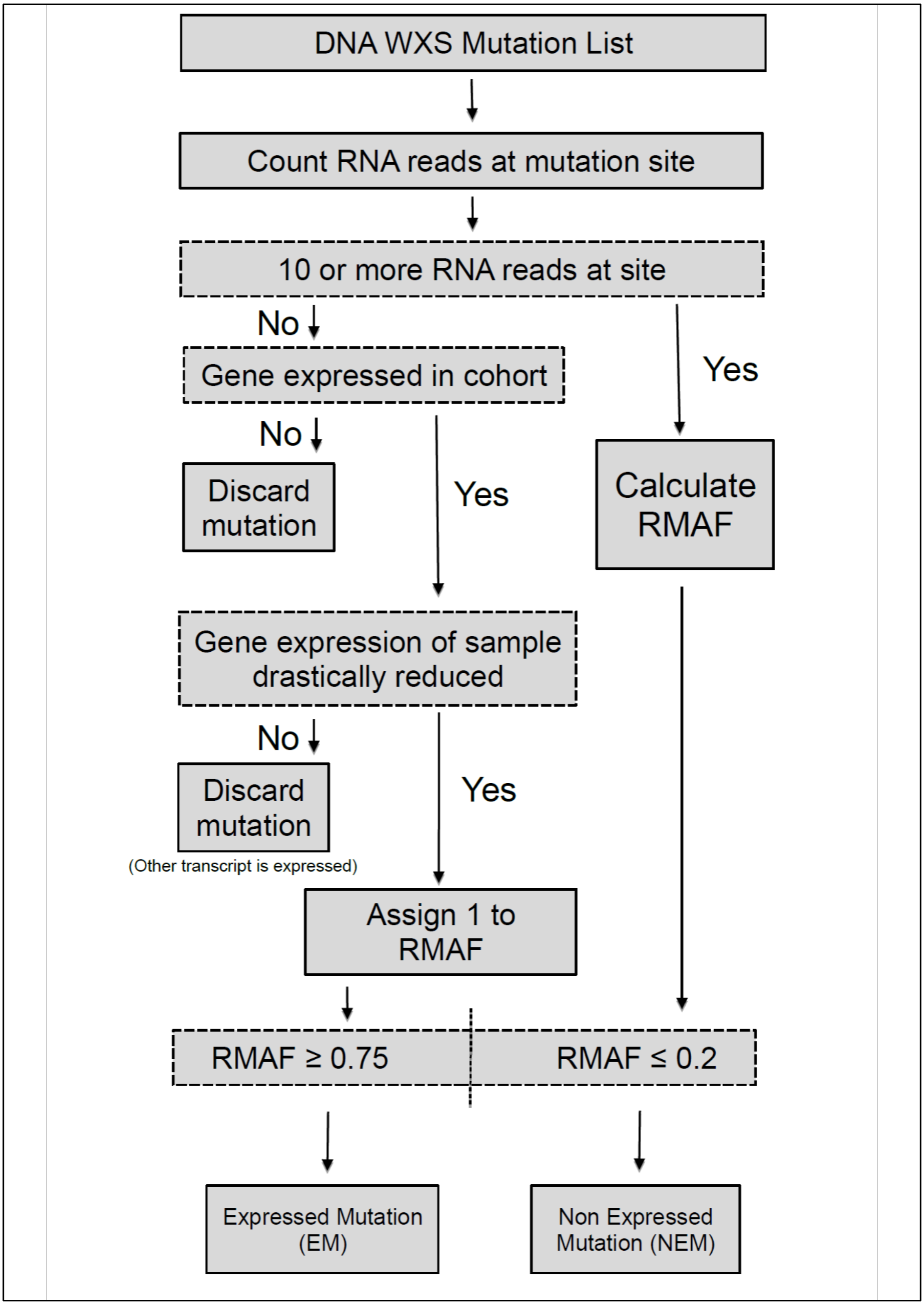

Supplementary Figure 6. Flow chart outlining the method for quantifying RNA Mutation Allele Frequency (RMAF). Mutations identified in the DNA Whole Exome Sequence (WXS) are quantitated in the mRNA. The level of expression dictates their classification as Expressed Mutations (EMs: RMAF ≥ 0.75) or Silent Mutations (SMs: RMAF≤ 0.2).

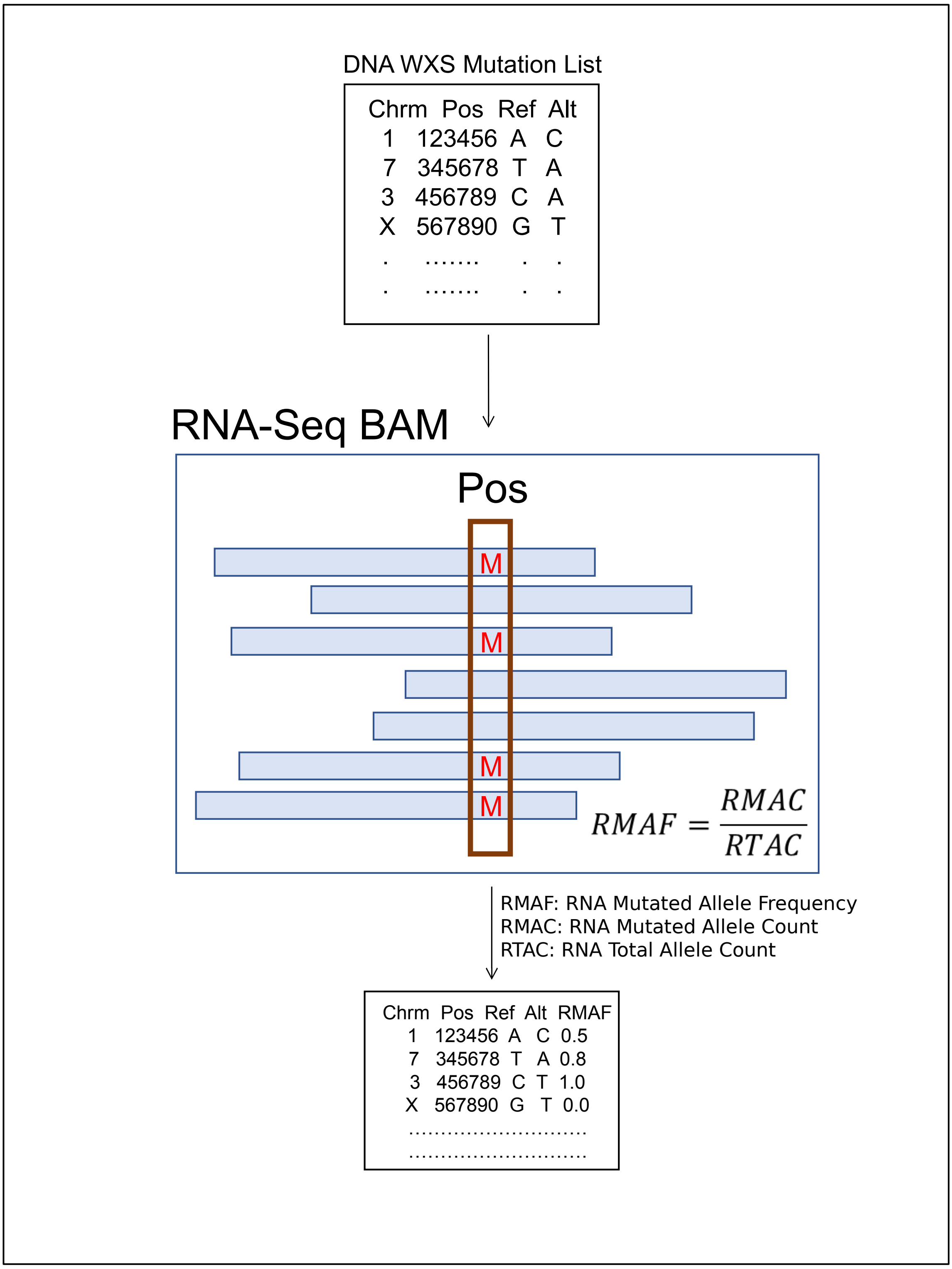

Supplementary Figure 7. RNA Mutated Allele Frequency (RMAF) is calculated from RNA Mutated Allele Frequency (RMAC) divided by RNA Total Allele Count (RTAC). Specific mutations in the DNA are quantitated in the RNA-seq data to determine the incidence of their frequency in mRNA. Four examples are given for four mucleotide positions on four different chromosomes, where the following abbreviations are used: Chrm (Chromosome), Pos (Position in the DNA), Ref (Reference sequence), Alt (Alternative sequence), M (mutated nucleotide).

Supplementary References

1. Olivier, M., Hollstein, M. & Hainaut, P. TP53 mutations in human cancers: origins, consequences, and clinical use. Cold Spring Harb Perspect Biol 2, a001008, doi:10.1101/cshperspect.a001008 (2010).
