# Supplementary material for: Identification of cancer sex-disparity in the functional integrity of p53 and its X chromosome network
Source: Nat Commun. 2019 Nov 26;10:5385. doi: 10.1038/s41467-019-13266-3 (PMC6879765; doi:10.1038/s41467-019-13266-3)
Supplement: Supplementary file 2 — Description of Additional Supplementary Files [file 41467_2019_13266_MOESM2_ESM.pdf]

Description of Additional Supplementary Files

File Name: Supplementary Data 1

Description: p53-STRING gene set analysis

TAB1. X-linked Genes that Interact with p53 as identified using the STRING Database (Version 10.5)*

TAB2. GO analysis of the p53-STRING gene set including TP53 in the analysis

TAB3. GO analysis of the p53-STRING gene set excluding TP53 in the analysis

File Name: Supplementary Data 2

Description: Analysis of Gene mutations in the non-reproductive cancers

TAB1: Number of mutations for each gene.

TAB2: Number of mutations for X-linked genes.

TAB3: Number of mutations for X-linked genes in females.

TAB4: Number of mutations for the p53-STRING genes in females.

TAB5: Number of mutations for X-linked genes in all males.

TAB6: Number of mutations for the p53-STRING genes in males.

TAB7: Summary of the numbers of X-linked gene mutation data.

File Name: Supplementary Data 3

Description: NEMs in the 12 disparity cancers

TAB1: Significance determination for NEMs among X-linked genes

TAB2: List of X-linked genes with significant NEMs

TAB3: GO analysis of X-linked genes with significant NEMs.

TAB4: T-test for NEMs among p53-STRING genes

TAB5: List of p53-STRING genes with significant NEMs

TAB6: GO analysis of p53-STRING genes with significant NEMs.

TAB7: References for p53-STRING genes with NEMs

TAB8: Analysis for NEMs in KIRC

File Name: Supplementary Data 4

Description: EMs in the 12 disparity cancers

TAB1: Significance determination for EMs among X-linked genes

TAB2: Significance determination for EMs among X-linked genes in females

TAB3: Significance determination for EMs among p53-STRING genes in females

TAB4: Significance determination for EMs among X-linked genes in males

TAB5: Significance determination for EMs among p53-STRING genes in males

TAB6: T-test for EMs among p53-STRING genes

TAB7: GO analysis of genes with significant EMs in males and females

File Name: Supplementary Data 5

Description: Analyses of Expression of Normal Samples

TAB1: GSEA analysis of X-linked negative regulators of p53

TAB2: Differential Expression of X-linked genes in TCGA normal samples

TAB3: Differential Expression of X-linked genes in 1000 Genomes Project
